# Applying Artificial Neural Networks to Oxidative Stress Biomarkers in Forager Honey Bees (*Apis mellifera*) for Ecological Assessment

**DOI:** 10.3390/toxics11080661

**Published:** 2023-08-01

**Authors:** Gianandrea La Porta, Gabriele Magara, Enzo Goretti, Barbara Caldaroni, Ambrosius Josef Martin Dörr, Roberta Selvaggi, Matteo Pallottini, Tiziano Gardi, Beniamino T. Cenci-Goga, David Cappelletti, Antonia Concetta Elia

**Affiliations:** 1 Department of Chemistry, Biology and Biotechnology, University of Perugia, 06126 Perugia, Italyenzo.goretti@unipg.it (E.G.); david.cappelletti@unipg.it (D.C.); 2 Department of Agricultural, Food and Environmental Sciences, University of Perugia, 06126 Perugia, Italy; 3 Department of Veterinary Medicine, University of Perugia, 06126 Perugia, Italy; beniamino.cencigoga@unipg.it

**Keywords:** biological indicators, biomarkers, environmental factors, metals, Self-Organizing Map

## Abstract

Insect pollinators provide an important ecosystem service that supports global biodiversity and environmental health. The study investigates the effects of the environmental matrix on six oxidative stress biomarkers in the honey bee *Apis mellifera*. Thirty-five apiaries located in urban, forested, and agricultural areas in Central Italy were sampled during the summer season. Enzyme activities in forager bees were analyzed using an artificial neural network, allowing the identification and representation of the apiary patterns in a Self-Organizing Map. The SOM nodes were correlated with the environmental parameters and tissue levels of eight heavy metals. The results indicated that the apiaries were not clustered according to their spatial distribution. Superoxide dismutase expressed a positive correlation with Cr and Mn concentrations; catalase with Zn, Mn, Fe, and daily maximum air temperature; glutathione S-transferase with Cr, Fe, and daily maximal air temperature; and glutathione reductase showed a negative correlation to Ni and Fe exposure. This study highlights the importance of exploring how environmental stressors affect these insects and the role of oxidative stress biomarkers. Artificial neural networks proved to be a powerful approach to untangle the complex relationships between the environment and oxidative stress biomarkers in honey bees. The application of SOM modeling offers a valuable means of assessing the potential effects of environmental pressures on honey bee populations.

## 1. Introduction

Insect pollinators provide an important ecosystem service by transferring pollen to crops and wild plants, thereby supporting global biodiversity and environmental health [1]. Spatial and temporal fluctuations in insect pollinator communities may have critical implications for conservation and agricultural production and constitute an important ecological challenge. Habitat loss and fragmentation, agrochemicals, persistent chemicals, airborne particulate matter, ozone, pathogens, alien species, and climate change are drivers of pollinator loss [2]. Indeed, pollinator decline may cause severe ecological and economic impacts that could negatively affect diversity and ecosystem stability, agricultural production, food security, and global economy [1,3]. 

Among the pollinators, honey bees, *Apis mellifera* Linnaeus, 1758, are eusocial insects living in large colonies of about 40,000 individuals. Colonies are composed of a single queen, a few males, and mostly females (workers), who are the smallest and sterile individuals, with a lifespan of about six weeks during the summer. Older workers are the forager bees that have the task of finding pollen, nectar, propolis, honeydew, and water.

Honey bees show good sensitivity to changes in environmental parameters and have the ability to accumulate contaminants in their tissues or in their products. Moreover, since all honey bees live together in nests or hives, they may come in contact with pollutants spread in the environmental compartments (air, soil, vegetation, and water) of an area commonly around 7 km^2^ surrounding their apiary [4]. For their ecological and biological traits, honey bees can record spatial and temporal pollutant variations playing a key role in the biomonitoring program of terrestrial environments [5]. Environmental contaminants arising from agricultural, urban, industrial activities, and changes in environmental parameters can impact organism survival. Pesticides, fungicides, insecticides, and heavy metals are considered key factors in the decline of bee colonies, affecting honey bees’ health [6].

Metals are a group of extremely heterogeneous contaminants spread widely in all environmental compartments, and their concentration is enhanced by anthropogenic activities. Metals can be transferred as particulate matter via the atmosphere or as inorganic/organic fertilizers and pesticides that contain heavy metal contaminants [7]. Considering their biological effects, some trace metals, such as lead (Pb), cadmium (Cd), and mercury (Hg), do not show any essential function for life and are toxic even in small quantities, whereas copper (Cu), zinc (Zn), and chromium (Cr), although essential at low levels, are very toxic at higher concentrations [8]. Chronic exposure to metals occurs through either direct exposure or trophic accumulation in the food web. Insects can bind both toxic (i.e., Pb, Sb, and Cd) and essential trace elements (i.e., Cu, Fe, and Zn) on the surface of the exoskeleton and/or incorporate them into body tissues [9]. Contaminant uptake can provide data about the bioavailable fraction that may provide evidence about the health condition of target organisms [10,11,12,13].

Honey bee colonies can be a useful tool in long-term metal monitoring, highlighting spatial and temporal variation of contaminant concentrations in a geographic area [5,14,15,16]. Nevertheless, it is important to note that contaminants such as heavy metals are not separately present in the environment, since contaminated sites often exhibit multiple valence states and complex compounds. In living organisms, such pollutants act as pro-oxidant forces, increasing the level of reactive oxygen species (ROS). ROS are a group of molecules containing at least one oxygen atom and one or more unpaired electrons (e.g., superoxide anion radical, hydroxyl radical, hydroperoxyl radical, and singlet oxygen). The defense mechanisms can be activated or overwhelmed by an excess of oxidizing compounds. Oxidative stress is the result of an imbalance between excessive cellular ROS production and antioxidant systems, including enzymatic and non-enzymatic molecules [17]. Superoxide dismutase is a metalloprotein with a redox active transition metal in the active site, ensuring the removal of anion superoxide through catalysis of its dismutation, producing H_2_O_2_. Catalase enzyme uses H_2_O_2_ as both oxidant and reductant, giving rise to the formation of water and O_2_, while glutathione peroxidase can reduce either hydrogen peroxide or organic peroxides. Glutathione reductase is a NADPH-dependent oxidoreductase that plays a key defense role against ROS. The enzyme-related detoxification mechanism regenerates GSH, the main scavenger of oxyradicals from GSSG. The multienzymatic family of glutathione S-transferases is a second defensive line against exogenic and endogenic molecules produced in ROS-mediated reactions. Acetylcholinesterase is the primary enzyme involved in the hydrolytic route of the neurotransmitter acetylcholine at cholinergic synapses.

Research has focused on examining stress biomarkers in honey bees to better understand the impact of anthropogenic activities on these insects [6,15,18,19,20]. Insect life-cycle timing [21,22,23] and antioxidant biomarkers can be affected by environmental stress [24,25,26], climate change [15], pesticides [27,28], and metals [29]. 

A previous study performed on the honey bee *A. mellifera ligustica* allowed us to outline a picture of metal contamination in the Umbrian region. The enrichment of Cd, Mn, Zn, and Mn in honey bees was related to the local characteristics along with the use of pesticides, fertilizers, the resuspension of the locally contaminated soils, and agriculture residues [7]. The present paper, based on the results of this previous study, investigated the effects of the environmental matrix on six oxidative stress biomarkers, such as superoxide dismutase, catalase, glutathione peroxidase, glutathione S-transferase, and glutathione reductase in the thorax-abdomen and acetylcholinesterase in the head of forager honey bees. We predicted that (i) metal bioaccumulation and the surrounding environmental conditions could affect the enzymatic response in bees, and (ii) enzymatic activities could provide a mathematical model to outline an apiary spatial pattern. We aimed to give toxicological information on bee colonies, which can be used for the management of bee colonies and for future comparative studies on ecological assessment of land types.

## 2. Materials and Methods

### 2.1. Honey Bee Sampling

**Figure 1:**
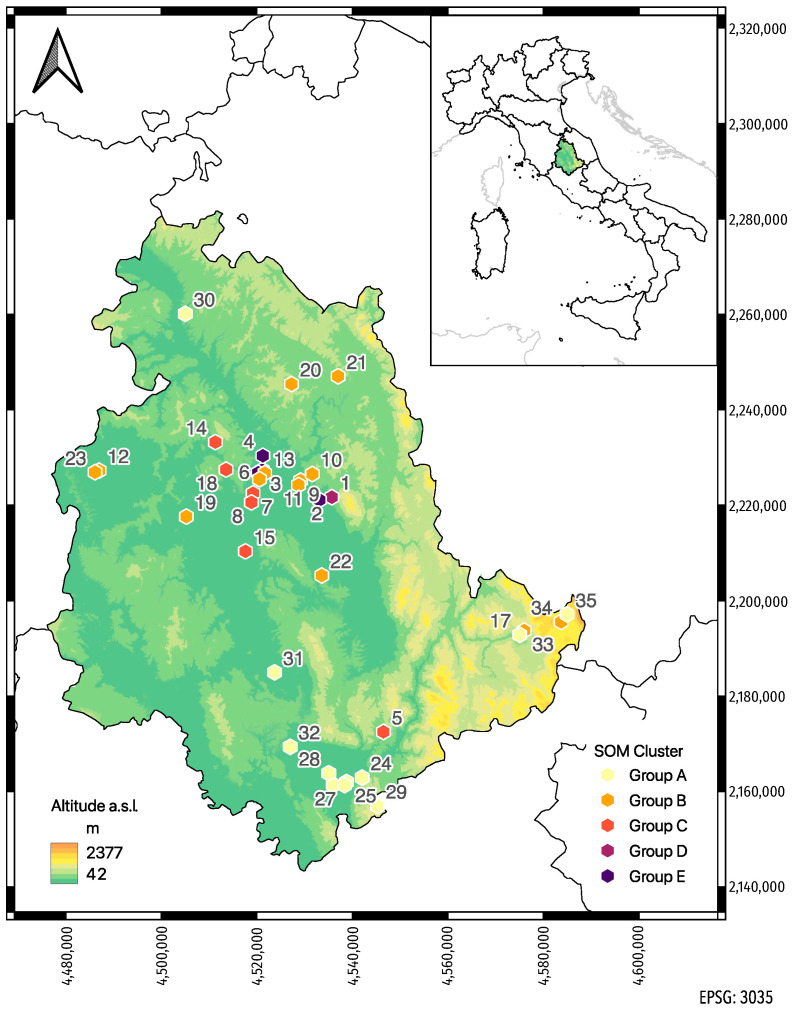
Location of sampling sites. Each apiary is labeled with an identification number from 1 to 35 and colored according to the cluster group to which it belongs in the Self-Organization Map (see Modeling Procedure and Statistical Analysis).

Thirty-five apiaries from various districts of the Umbria Region, Central Italy, were investigated during summer 2014 and 2015 (Figure 1).

Sampling sites were selected by considering different degrees of environmental pollution mainly related to agricultural and industrial activities (for more detailed information see [7,30]). All samplings were carried out with the beekeepers’ consent to participate anonymously in the present study. Honey bee samplings were carried out according to safety rules and without opening the hives and disturbing the activity of the insects. For each apiary, a single central hive was selected as a representative sample of the sampling site. We placed a plastic bag at the hive entrance and collected a sample of about 100 forager bees. Indeed, under undisturbed conditions, bees outgoing from hives are generally those designated for daily foraging activities. The purpose of selecting summer foragers was to minimize the influence of seasonal conditions, age, and polyethism by enhancing the relevance of potential biomarker responses and the effects of the environmental matrix.

Honey bee samples were refrigerated on site (4 °C), and then immediately transferred and stored in the laboratory at −80 °C, in order to guarantee the correct preservation of samples. Before being submitted to the analytical procedures, each honey bee specimen was cleaned from pollen and possible parasites, i.e., Varroa destructor (Anderson and Trueman, 2000), and then some keratinized parts, such as wings and legs, were removed. The thorax-abdomen and head of each specimen were dissected, placed in labeled test tubes, and stored at −80 °C. These specimens were utilized in biochemical analyses for the evaluation of oxidative stress biomarkers.

### 2.2. Oxidative Stress Biomarkers

The antioxidant biomarker activity of superoxide dismutase (SOD), catalase (CAT), glutathione peroxidase (GPx), glutathione reductase (GR), and glutathione S-transferase (GST) was investigated in the cytosolic fraction of the thorax-abdomen and acetylcholinesterase (AChE) levels in the head of each specimen according to published procedures [12]. Bees collected from each site for biomarker analysis were divided into 6 similarly weighted pools of 2 individuals. Each group was then analyzed individually for oxidative stress biomarkers. Tissues were homogenized (1:5) in a 100 mM KP buffer, sodium chloride (NaCl) 2.5%, 0.1 mg/mL bacitracin, and 0.008 TIU/mL aprotinin and centrifuged for 15 min at 21.000× *g*. Biomarkers were measured in triplicate by spectrophotometry (Varian Cary 50) at 25 °C. All biochemical analyses of enzyme levels were normalized to the protein concentration [31].

SOD (E.C. 1.15.1.1, IUBMB 1992 [32]) activity was evaluated in 50 mM Na_2_CO_3_ buffer pH 10 with 0.1 mM EDTA, 500 mM cytochrome C, 1 mM hypoxanthine, and xanthine oxidase. Cytochrome C reduction by the xanthine/hypoxanthine complex was evaluated by comparison with a standard SOD unit curve hypoxanthine, and xanthine oxidase at 550 nm. CAT (E.C. 1.11.1.6) activity was measured at 240 nm after the decrease in absorbance following the consumption of H_2_O_2_. The assay was carried out in NaH_2_PO_4_ buffer + Na_2_HPO_4_ 100 mM pH 7 and H_2_O_2_ 24 mM. GPx (E.C. 1.11.1.9) activity was determined at 340 nm in NaH_2_PO_4_ + Na_2_HPO_4_ 100 mM buffer, pH 7.5, 1 mM EDTA, 0.12 mM NADPH (β-nicotinamide adenine dinucleotide), 2 mM GSH, 1 U of GR (glutathione reductase), 1 mM NaN_3_, and 0.6 mM H_2_O_2_. GST (E.C. 2.5.1.18) activity was assessed at 340 nm in NaH_2_PO_4_ + Na_2_HPO_4_ 100 mM pH 6.5 buffer with GSH and CDNB (1-chloro-2,4-dinitrobenzene) 2 mM. GR (E.C. 1.6.4.2) activity was measured at 340 nm in NaH_2_PO_4_ + Na_2_HPO_4_ 100 mM buffer, pH 7, 1 mM GSSG (oxidized glutathione), and 0.06 mM NADPH. AChE (E.C. 3.1.1.7) activity was measured according to the method of [33], slightly modified, measuring the increase in absorbance at 412 nm in 100 mM NaH_2_PO_4_ + Na_2_HPO_4_ pH 7.5 buffer, 1 mM 5,5’-dithiobis-2-dinitrobenzoic acid (DTNB), and 2 mM acetylthiocholine.

### 2.3. Modeling Procedure

A buffer zone of 1500 m was defined around the beehives to identify the prevalent land use type in the foraging zones of bee colonies. Based on the varying percentages of land use types, three primary land types were identified: natural, rural, and urban areas. Natural and rural categories were further divided into two subcategories based on the level of urban infrastructure in the area, resulting in a total of five land type classes coded as follows: 1—natural areas; 2—natural areas with urban sprawl and olive groves; 3—rural areas with low urban infrastructure; 4—rural areas with moderate urban infrastructure; and 5—urban areas.

Moreover, information on ground temperature and precipitation in the surrounding areas was collected from the hydro-pluviometric and thermometric monitoring station system of the Umbria Region SIR (http://www.annali.regione.umbria.it/, accessed on 1 April 2023). For each apiary, the nearest monitoring station was selected to collect data on the average daily maximum air temperature (°C) and total rainfall (mm) for the month in which the bees were sampled. Metal accumulation levels were taken from Goretti et al. [7]. The Moran I test was used to evaluate spatial autocorrelation by quantifying the level of similarity or dissimilarity among neighboring observations.

An artificial neural network (ANN) was used to examine the impact of the environmental matrix on several oxidative stress biomarkers in bees. The ANN was a Self-Organizing Map (SOM), an unsupervised neural network useful for pattern recognition. This algorithm is commonly utilized for dimensionality reduction and for exploring linear and nonlinear relationships within high-dimensional datasets [34]. In fact, it involves the creation of a series of neurons, commonly referred to as nodes, that minimize their distances and organize themselves into a bidimensional map that optimizes the topology to maintain the original input data relationships. As a result, nearby unit nodes share similar associated characteristics. This approach was applied to uncover the existence of clusters in the apiary locations based on the oxidative stress levels of the bees. The SOM neural network was composed of two layers of neurons: (i) the first was the input layer connected to enzymatic values of bees in different apiaries and (ii) the second consisted of the output neurons, which represent the map. Moreover, the nodes, also known as “proto-cluster” [35], were further grouped in clusters to generate a quantitative description of data derived from biochemical analyses. In other words, we used a two-level approach: initially, the enzymatic activities were clustered using the SOM; subsequently, the nodes of the SOM were clustered using the k-means clustering algorithm. The organization and visualization of the SOM were carried out using the R package “kohonen” [36].

### 2.4. Statistical Analysis

To understand how oxidative stress in bees is affected by environmental factors and metal accumulation levels, the method proposed by Park et al. [37] was followed. The objective of the analysis was to integrate the environmental and metal accumulation data into the Self-Organizing Map (SOM) developed to categorize the apiaries according to their oxidative stress levels.

The analysis was carried out in four steps: 1. The bee apiaries were assigned to different nodes using the trained Self-Organizing Map (SOM); 2. For each node on the SOM, the median values of each environmental and metal accumulation variable of the assigned apiaries were calculated. Previously, the metal accumulation levels were log-transformed to normalize their distribution and reduce skewness; 3. The median values obtained in step 2 were represented in a new grayscale map, where the nodes and their corresponding apiaries kept their relative positions according to the trained SOM in steps 1 and 4. Rank correlation and regression analyses were performed between the nodes on the SOM and the median values of the metal and environmental variables. This final step aimed to examine the relationships and associations between the clusters of apiaries and the specific environmental and metal accumulation characteristics.

A significance value of 0.05 was used to determine whether the relationships observed were statistically meaningful. All the statistical analyses were conducted using the R statistical framework [38] within the RStudio integrated development environment [39].

## 3. Results

### 3.1. Biomarker Activities

The antioxidant biomarker activities recorded in forager bees exhibited percentage variations from the minimum to the maximum value ranging from 131 to more of the 1000-fold, with either not significant or negative spatial autocorrelations (Table 1).

### 3.2. Self-Organizing Map

**Figure 2:**
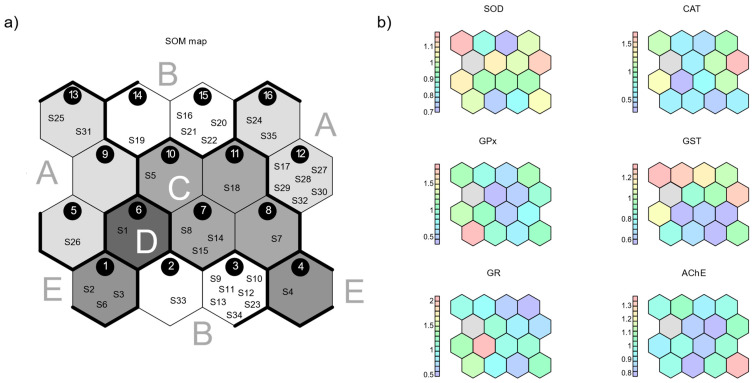
(**a**) Classification of the 35 apiaries through the training of 4 × 4 SOM with 6 oxidative stress biomarkers. Dark edges and shades of gray highlight the cluster groups labeled with the letters A–E. (**b**) Codebook vectors of each node for a single biomarker. The legend refers to scaled values. Empty units are depicted in gray.

Biomarker activities, appropriately scaled to remove the effect of their different dimensionalities, guided the learning process of a Self-Organizing Map (SOM) with a hexagonal-toroidal topology consisting of 16 nodes. This resulted in the arrangement of the 35 apiaries following the learned patterns (Figure 2a). Hexagon nodes contained from a minimum of 1 to a maximum of 7 apiaries, with the only exception being empty node number 9. The trained map presented an average distance between each apiary and the respective closest unit in the map of 0.13 (sd = 0.09), with an efficiency in the topological representation of 0.91. Results from the SOM and the following k-means analysis showed the existence of five main clusters among the bee apiaries based on the level of the oxidative stress biomarkers. The apiaries in group or cluster A were characterized by elevated values of GST, CAT, and SOD; those of group B exhibited low values for all the biomarkers except for GST; group C showed moderate levels of SOD and minimal activities of GPx and AChE; in group D, high values of GR were observed with almost no activity for GST; lastly, group E showed intermediate values, with some apiaries having high levels of GPx or AChE (Figure 2b).

**Figure 3:**
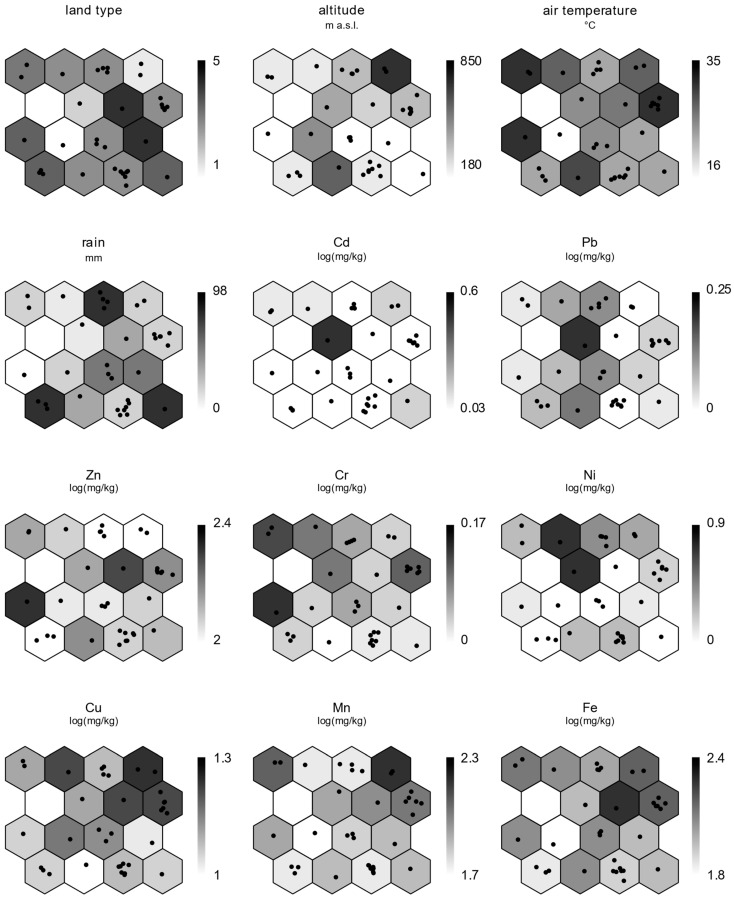
Environmental factors and bioaccumulation levels in the SOM map. Black dots in the SOM nodes represent the apiaries. Land type: 1—Natural areas; 2—Natural areas with urban sprawl and olive groves; 3—Rural areas with low urban infrastructure; 4—Rural areas with moderate urban infrastructure; 5—Urban areas.

Each node was also inspected in relation to the environmental conditions surrounding the apiaries and bioaccumulation levels of the metals in forager bees (Figure 3). Darker shades of gray indicate nodes with higher values, while those with lighter shades represent nodes with lower values. The nodes in group A exhibited a heterogeneous environmental situation, where rural areas and elevated air temperature predominated. Bees had high levels of Fe, Mn, and Cr. The nodes in group B showed elevated air temperature, and apiaries were mainly located in rural areas with low urbanization levels. Bees had high levels of Ni, Cu, and, in a single case, Pb. The nodes in group C were characterized by apiaries mainly situated in urban or suburban areas at low altitudes; their bees had levels of Fe, varying from intermediate to high. Additionally, bee samples from node 10 of this group had the highest levels of Cd, Ni, and Pb. Group D had a single apiary located in a natural area and exhibited low values for all metals except Cu. Group E, including apiaries located in the rural areas close to the urban centers, was characterized by high levels of rainfall and showed low accumulation values of metals.

### 3.3. Correlations

**Figure 4:**
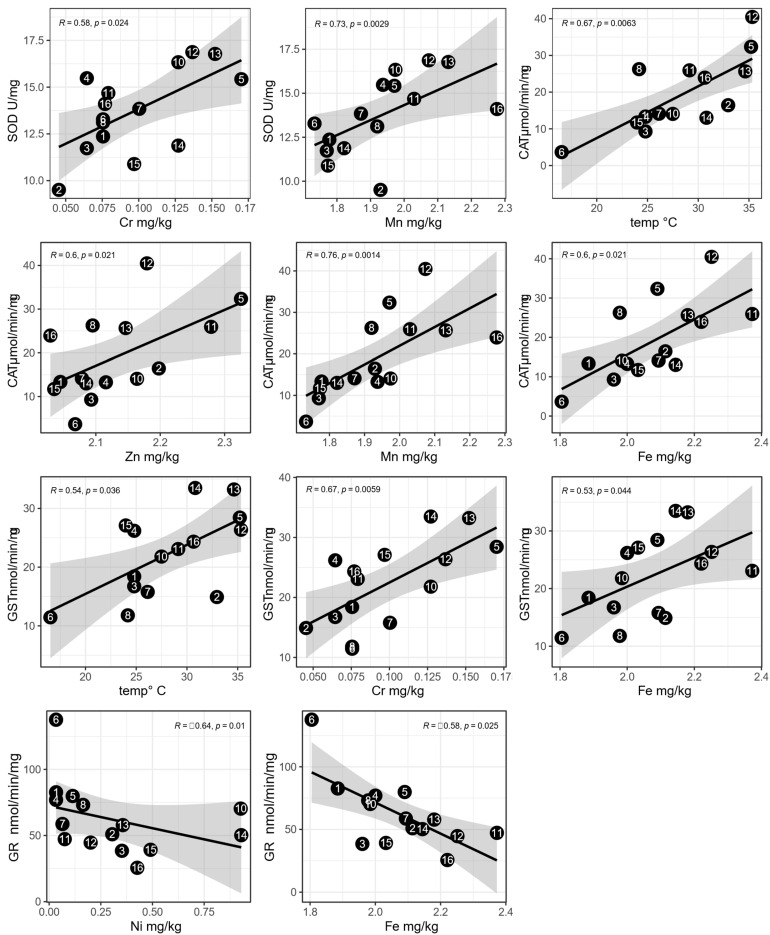
Regression and correlations between environmental parameters or metal accumulation levels and biomarkers (median value of the node). The numbered black dots correspond to the nodes in the SOM map. Only statistically significant correlations (*p* < 0.05) are reported along with the correlation coefficients (*R*).

The correlation and regression analyses revealed that SOD, CAT, GST, and GR showed moderate to strong statistically significant relationships with five out of eight metals and one out of four environmental parameters (Figure 4) (Table S1).

Specifically, it was recorded that SOD expressed a positive trend with Cr and Mn; CAT with Fe, Mn, Zn, and daily maximum air temperature; GST with Cr, Fe, and daily maximum air temperature; and finally, GR showed a negative correlation with Fe and Ni. It is worth noting that, even for environmental variables, spatial autocorrelation showed either not statistically significant or negative values (Table S2).

## 4. Discussion

The present study investigated the pattern of oxidative stress in honey bees, considering both metal bioaccumulation levels and the potential effects of selected environmental factors. Herein, oxidative stress biomarkers in bee tissues were correlated with land type, altitude, mean monthly values of maximum temperature, mean monthly rainfall, and body metal accumulation recorded for each apiary [7].

The SOM analysis indicated that the apiaries were not clustered according to their spatial distribution. Likewise, oxidative stress biomarkers were not strongly related to the macro-scale spatial position of the apiaries. These outcomes are consistent with those of the autocorrelation analysis, which showed either an absence of autocorrelation or a negative autocorrelation for biomarkers. Even the type of land use surrounding the apiaries did not show a statistically significant correlation with enzyme activity. Therefore, other factors may be more important in determining the different levels of oxidative stress biomarkers in honey bees from Umbrian apiaries. Four out of six biomarkers (SOD, CAT, GST, and GR) showing a relationship with the levels of temperature and metals such as Cr, Mn, Zn, Fe, and Ni in the tissues of forager bees are discussed here. The correlations between GPx and AChE in honey bees, considering environmental and stress factors, were not found to be statistically significant. This indicates that there were no clear patterns or nonlinear trends observed between these variables. Hence, these relationships need to be further explored in the future using different datasets.

Many pollinators are found in severely metal-altered land types; however, for their biological traits, it is difficult to ascertain the chronic metal contamination of an exact site [40]. Deepening the knowledge of how environmental pollutants along with other stressors (e.g., temperature changes and reduced habitat quality) influence pollinators’ health is required for conservation planning [40].

Bee exposure to metals is known to be linked to local farming practices, including the usage of pesticides and fertilizers, but it is also connected to airborne metal content. Indeed, elevated concentrations of Cr, Cu, As, Cd, Al, and Zn can be found in urban soils near former industrial and waste disposal, as well as in landscapes far away from a metal point source [40]. As already highlighted by Goretti et al. [7], enrichment of Cd, Zn, Cu, and Mn in honey bees was related to metal contamination occurring in different Umbrian land types. In fact, the Umbrian dataset showed that most of the sites fell within values of low and intermediate metal contamination compared with data recorded for other national and European regions. Nevertheless, the heterogeneous territory of the Umbria region and the occurrence of metals in urbanized or agricultural sites may impact organism health or their trophic networks [7,41]. Antioxidant enzymatic activities may be reliable indicators of contaminants’ impact even before their effects on survival, life cycle, fecundity, or dispersal of honey bees [18,19,42]. The co-occurrence of several stressors in habitats makes it difficult to ascertain the effect of a single metal on the physiological pathways. For instance, a laboratory study underlined that the fitness of honey bee larvae and foragers of *Apis mellifera* was reduced under co-exposure (1:1) of Cd and Cu [42].

Previous studies highlighted that honey bees from more anthropized areas showed a boost in detoxification processes, likely due to the occurrence of environmental pollutants [27,29]. In particular, the oxidative stress biomarkers in honey bees were related to metal contamination in different territories; bees from urban and industrial areas had increased expression of both Sod1 and Cat genes, although the inhibitory effect of high Pb bioaccumulation on CAT was recorded in specimens from industrial areas [29]. Beetles from metal-polluted sites showed different enzyme responses, likely due to their higher polymorphism of antioxidant enzymes [43].

Metal-induced oxidative stress involves complex milieus in organisms. Redox-active metals, such as Fe, Cr, and Cu, generate ROS through redox cycling or are involved in the Fenton route. Negative side effects include the rise in ROS concentration, which is documented as a promoter of metal toxicity [11]. Chromium is a metal with changeable valence and can undergo the Haber-Weiss reaction. Among the chromium species, Cr (VI) is generally more mobile, soluble, and toxic than Cr (III). However, Cr (III) in trace amounts is an essential element of human and animal nutrition and is the dominant form occurring in soils [44]. A reduction from Cr (VI) to Cr (III) may occur in soils because of their high organic matter content. Cr reduction coupled with O_2_ activation triggers a high oxygen radical level [45]. Cellular localization and interconversion of Cr speciation, such as oxidization of Cr (III) to Cr (VI), promotes toxicity in metal-exposed organisms.

The catalytic activity and gene expression of the different isoforms of SOD can vary under stressful conditions in honey bees [29]. Herein, we measured Cu/Zn-SOD activity, the isoform mainly found in cytosol of eukaryotes. Overproduction of –OH concentration can severely disturb enzyme function via oxidization of histidine, one of the Cu ligands, thus releasing Cu. Even Zn may trigger a mechanism by which Cu can be replaced in the SOD subunits. Although Mn is a cofactor for several enzymes, both excessive and insufficient metal exposure can elicit shifts in metal homeostasis, resulting in a multifaceted effect on organisms. The enhancement of SOD activity recorded in metal-exposed honey bees suggests a compensatory mechanism against ROS. Indeed, in the present study, SOD activity in specimens from higher Cr and Mn polluted apiaries plays a key role in reducing the toxicity of such metals and protects bees against oxidative stress pressure.

Higher oxidative stress (lipid peroxidation) was recorded in feral foragers compared with the managed colonies of honey bees. According to the authors, the outcome suggested a tolerance mechanism rather than a repair mechanism to survive [46]. Similarly, Li-Byarlay et al. [47] suggested an oxidative stress tolerance in drones (males) experimentally exposed to herbicide methyl viologen, also known as “paraquat”. Longer-lived males showed higher levels of malondialdehyde than early-dying ones [47]. Moreover, over-expression levels of CYP9Q1, CYP9Q2, CYP9Q3, and genes encoding SOD and CAT were related to higher Hg and Pb pressure in *Apis mellifera* collected from urban zones than those from agricultural and mountainous areas [48].

Oxidative stress is one of the physiological costs associated with the flight activity of honeybees. A likely increase in ROS level in flight muscle, coupled with age-related decreases in oxidative stress biomarkers, may be responsible for behavioral senescence and reduced longevity [49]. Enhanced SOD levels can be a key strategy for offsetting ROS load produced during the foraging efforts of insects. Indeed, SOD1-deactivated insects have shortened lifespans due to their inability to counterbalance ROS toxicity [50].

Catalase is a widespread enzyme in various organelles of insect cells [43]. Iron-sulfur clusters of CAT require Fe as an essential element in its active center. Normal physiological conditions ensure a dynamic balance between both enzymes SOD and CAT in removing free radicals. The quantitative dynamic relationship between both enzymes in honey bees sampled from Umbrian apiaries was not disturbed by Zn, Mn, and Fe exposure. Zinc is an essential element involved in several cellular routes as an antioxidant factor. Although Zn is a trace metal naturally present in the environment, anthropogenic activities can raise its concentration [51]. However, as underlined above for Mn, both excessive and insufficient metal exposure may elicit many side effects on organisms. In the present study, among the tested biomarkers, only the CAT enzyme showed slightly higher activity in bees from urban areas compared to those collected from natural areas. However, Nicewicz et al. [52] showed no differences in levels of GST and AChE in several tissues (brain, fat body, thorax muscle, and gut) of foragers *A. mellifera* collected from an urban and a rural apiary; low levels of PM10, PM2.5, As, and benzo-α-pyrene was reported in the latter. According to the authors, only heat shock proteins, defensin, and total antioxidant capacity in fat can be useful biomarkers in detecting urban multi-stress factors [52].

Indeed, in the present study, the enhanced GST activity in the apiaries of groups A and B denotes an important outcome, since this indicates elevated phase-II biotransformation metabolism. Strengthening the detoxification pathways allows bees to better offset the pressure of environmental stressors. Even changes in temperatures may enhance ROS levels in organisms, disturbing their physiological homeostasis. Honey bees are poikilothermal animals: air temperatures below 7 °C or above 38 °C may affect the metabolic and physiological responses, thereby also inhibiting their foraging [5,26]. Particularly, high values of temperature and humidity upregulate cytochrome P450 (cytP450), as well as heat shock proteins (Hsp), triggering oxidative stress in bees [53]. Furthermore, diverse proteotoxic stresses can activate the expression of genes encoding small heat shock proteins (sHSP) of the *lethal (2) essential for life (l(2) ef) gene family* [54].

Herein, nodes of both groups A and B were associated with high values of air temperature along with certain trace (Fe, Mn, and Cu) and heavy metal (Cr, Ni, and Pb) levels in bees. An increase in temperature-related CAT and GST activity in the same nodes denotes a strengthening of the detoxifying ability necessary to counteract the pro-oxidant effects induced by pollutants. Moreover, GSTs play an important role in the overall fitness of insects exposed to pollutants. High levels of lipid peroxidation products subsequent to changes in environmental temperatures can be metabolized by GSTs. Increased expression of the zeta class GST (AccGSTZ1) was recorded in *A. cerana cerana* under temperature challenges and H_2_O_2_ [55]. Honey bees can offset heat stress through upregulation of several key genes and proteins, such as acetylcholinesterase 1 (AchE1) and Zn finger protein, reducing ROS content and likely increasing their survival rate [56].

We noted a Ni- and Fe-related pattern of GR activity in bees, reflecting a weakening of defense mechanisms against excess oxidizing compounds. In particular, the nodes of group B showed high levels of Ni, whereas those of group C (located in urban or suburban areas at low altitudes) had intermediate to high levels of Fe, along with the lowest GR activity. Nickel belongs to transition metals that readily contribute to oxidoreductive reactions, and its toxicity is partially associated with the formation of free radicals. Changes in GR activity may also trigger low levels of GSH, which is one of the most powerful antioxidant molecules ubiquitously distributed through organisms. The reduced thiol can promote oxidative defense directly or as a cofactor or substrate of antioxidant enzymes, such as GPx and GST. High O_2_^−^ concentration may produce oxidation of the –SH groups, reducing the enzymatic activity. Therefore, ROS enhancement, along with lowered GR activity, may promote Ni and Fe toxicity in bees. Although most studies examining how exposure to common soil contaminants (Cd, Pb, Cu, and Zn) within many legacy cities influence pollinator health, As, Cr, Ni, and Fe can also be recurrently found at elevated levels, severely triggering insect pollinators [40]. Indeed, the altered antioxidant biomarker levels may be recognized as early metal-mediated injury in honey bees.

## 5. Conclusions

Although the dataset of the present study is limited to a regional area, the artificial neural network (ANN) is confirmed to be a powerful approach. This statistical method can clarify and summarize the relationships between the environmental matrix and the oxidative stress biomarkers in honey bees. Future studies employing SOM modeling offer a promising avenue for advancing our comprehension of the effects of environmental stressors on honey bees, ranging from regional to large-scale contexts, and dealing with both simple and complex scenarios.

## Figures and Tables

**Table 1:** Summary statistics of oxidative stress biomarkers of the pooled samples.

Biomarker	n	Min	Max	Median	iqr	Mean	SD	95% CI	% Variation	Spatial Autocorrelation
SOD	35	6.10	18.31	13.28	4.16	13.57	2.86	0.98	200%	−0.113 (*p* < 0.001)
CAT	35	3.67	45.02	14.12	14.45	19.81	11.80	4.05	1128%	−0.185 (*p* < 0.001)
GPx	35	3.51	31.98	12.90	8.15	14.26	6.57	2.26	811%	−0.020 (*p* = 0.620)
GST	35	10.73	40.91	23.01	10.89	22.36	8.13	2.79	281%	−0.095 (*p* = 0.001)
GR	35	14.96	137.74	49.29	24.71	52.05	24.53	8.43	821%	−0.050 (*p* = 0.296)
AChE	35	45.11	104.35	65.97	13.33	65.93	13.41	4.61	131%	−0.010 (*p* = 0.342)

## Data Availability

Data will be made available on request.

## Supplementary Materials

The following supporting information can be downloaded at: https://www.mdpi.com/article/10.3390/toxics11080661/s1, Table S1. Moran’s I autocorrelation coefficients for the environmental matrix; Table S2. Correlation coefficients (r) between oxidative stress biomarkers and environmental variables.
