# Supplementary material for: Applying Artificial Neural Networks to Oxidative Stress Biomarkers in Forager Honey Bees (Apis mellifera) for Ecological Assessment
Source: Toxics. 2023 Aug 1;11(8):661. doi: 10.3390/toxics11080661 (PMC10459414; doi:10.3390/toxics11080661)
Supplement: Supplementary file 1 [file toxics-11-00661-s001.zip › Table S1.pdf]

Applying Artificial Neural Networks to Oxidative Stress Biomarkers in Forager Honey Bees (*Apis mellifera*) for Ecological Assessment

Gianandrea La Porta [1,*,†], Gabriele Magara [1,†], Enzo Goretti [1], Barbara Caldaroni [1], Ambrosius Josef Martin Dörr [1], Roberta Selvaggi [1], Matteo Pallottini [1], Tiziano Gardi [2], Beniamino T. Cenci-Goga [3], David Cappelletti [1] and Antonia Concetta Elia [1,*]

Table S1. Moran's I autocorrelation coefficients for environmental matrix.

**variable**	**spatial autocorrelation**	**p.value**
land type	-0.067	0.064
altitude	-0.158	>0.001
air temperature	-0.246	>0.001
total rainfall	-0.151	>0.001
Zn	-0.075	0.024
Cr	-0.178	>0.001
Cu	-0.081	0.011
Mn	-0.103	>0.001
Fe	-0.108	>0.001
Cd	-0.042	0.472
Pb	-0.020	0.618
Ni	-0.044	0.481
