# Supplementary material for: Applying Artificial Neural Networks to Oxidative Stress Biomarkers in Forager Honey Bees (Apis mellifera) for Ecological Assessment
Source: Toxics. 2023 Aug 1;11(8):661. doi: 10.3390/toxics11080661 (PMC10459414; doi:10.3390/toxics11080661)
Supplement: Supplementary file 1 [file toxics-11-00661-s001.zip › Table S2.pdf]

# Applying Artificial Neural Networks to Oxidative Stress Biomarkers in Forager Honey Bees (*Apis mellifera*) for Ecological Assessment

**Gianandrea La Porta [1,*,†], Gabriele Magara [1,†], Enzo Goretti [1], Barbara Caldaroni [1], Ambrosius Josef Martin Dörr [1], Roberta Selvaggi [1], Matteo Pallottini [1], Tiziano Gardi [2], Beniamino T. Cenci-Goga [3], David Cappelletti [1] and Antonia Concetta Elia [1,*]**

**Table S2.** Correlation coefficients (r) between oxidative stress biomarkers and environmental variables.

| var1 | var2 | r | p |
|---|---|---|---|
| SOD | land type | 0.098 | 0.728 |
| SOD | altitude | -0.100 | 0.714 |
| SOD | temperature | 0.450 | 0.091 |
| SOD | rain | -0.370 | 0.172 |
| SOD | Cd | 0.450 | 0.097 |
| SOD | Pb | -0.310 | 0.262 |
| SOD | Zn | 0.420 | 0.119 |
| SOD | Cr | 0.580 | 0.024 |
| SOD | Ni | -0.120 | 0.675 |
| SOD | Cu | 0.390 | 0.148 |
| SOD | Mn | 0.730 | 0.003 |
| SOD | Fe | 0.360 | 0.192 |
| CAT | land type | 0.450 | 0.094 |
| CAT | altitude | -0.140 | 0.611 |
| CAT | temperature | 0.670 | 0.006 |
| CAT | rain | -0.280 | 0.314 |
| CAT | Cd | -0.089 | 0.751 |
| CAT | Pb | -0.300 | 0.283 |
| CAT | Zn | 0.600 | 0.021 |
| CAT | Cr | 0.450 | 0.093 |
| CAT | Ni | -0.036 | 0.899 |
| CAT | Cu | 0.046 | 0.873 |
| CAT | Mn | 0.760 | 0.001 |
| CAT | Fe | 0.600 | 0.021 |
| GPx | land type | 0.110 | 0.703 |
| GPx | altitude | -0.021 | 0.944 |
| GPx | temperature | 0.230 | 0.403 |
| GPx | rain | -0.020 | 0.945 |
| GPx | Cd | 0.079 | 0.781 |
| GPx | Pb | -0.360 | 0.184 |

| | | | |
|---|---|---|---|
| GPx | Zn | -0.120 | 0.676 |
| GPx | Cr | -0.032 | 0.909 |
| GPx | Ni | -0.310 | 0.264 |
| GPx | Cu | -0.200 | 0.466 |
| GPx | Mn | 0.140 | 0.611 |
| GPx | Fe | -0.075 | 0.793 |
| GST | land type | 0.140 | 0.617 |
| GST | altitude | -0.014 | 0.964 |
| GST | temperature | 0.540 | 0.036 |
| GST | rain | -0.450 | 0.096 |
| GST | Cd | 0.077 | 0.785 |
| GST | Pb | -0.260 | 0.351 |
| GST | Zn | 0.150 | 0.602 |
| GST | Cr | 0.670 | 0.006 |
| GST | Ni | 0.460 | 0.085 |
| GST | Cu | 0.270 | 0.320 |
| GST | Mn | 0.410 | 0.130 |
| GST | Fe | 0.530 | 0.044 |
| GR | land type | 0.250 | 0.361 |
| GR | altitude | -0.390 | 0.152 |
| GR | temperature | -0.260 | 0.353 |
| GR | rain | 0.190 | 0.507 |
| GR | Cd | 0.190 | 0.507 |
| GR | Pb | 0.310 | 0.262 |
| GR | Zn | 0.120 | 0.667 |
| GR | Cr | -0.039 | 0.889 |
| GR | Ni | -0.640 | 0.011 |
| GR | Cu | -0.460 | 0.086 |
| GR | Mn | -0.260 | 0.354 |
| GR | Fe | -0.580 | 0.025 |
| AChE | land type | 0.020 | 0.943 |
| AChE | altitude | -0.270 | 0.327 |
| AChE | temperature | -0.300 | 0.276 |
| AChE | rain | 0.047 | 0.869 |
| AChE | Cd | 0.410 | 0.130 |
| AChE | Pb | -0.270 | 0.337 |
| AChE | Zn | -0.340 | 0.216 |
| AChE | Cr | -0.130 | 0.642 |
| AChE | Ni | -0.025 | 0.929 |
| AChE | Cu | 0.064 | 0.822 |
| AChE | Mn | -0.250 | 0.375 |
| AChE | Fe | -0.270 | 0.320 |
